# A Sustainable Technique to Prepare High-Purity Vanadium Pentoxide via Purification with Low Ammonium Consumption

**DOI:** 10.3390/ma15051945

**Published:** 2022-03-05

**Authors:** Guoce Lin, Jing Huang, Yimin Zhang, Pengcheng Hu

**Affiliations:** 1 School of Resource and Environmental Engineering, Wuhan University of Science and Technology, Wuhan 430081, China; guocelin@163.com (G.L.); zym126135@126.com (Y.Z.); hpcmy126@126.com (P.H.); 2 State Environmental Protection Key Laboratory of Mineral Metallurgical Resources Utilization and Pollution Control, Wuhan 430081, China; 3 Collaborative Innovation Center of Strategic Vanadium Resources Utilization, Wuhan 430081, China; 4 Hubei Provincial Engineering Technology Research Center of Highly Efficient Cleaning Utilization for Shale Vanadium Resource, Wuhan 430081, China

**Keywords:** V_2_O_5_, vanadium precipitation, replacement, purification

## Abstract

The general preparation method for V_2_O_5_ is ammonium salt vanadium precipitation, which inevitably produces large amounts of ammonia nitrogen wastewater. In this paper, we propose an environmentally friendly method for preparing high-purity V_2_O_5_ with low ammonium consumption. The purity of the V_2_O_5_ product reaches more than 99% while reducing the level of ammonium consumption. The vanadium precipitation efficiency reaches 99.23% and the V_2_O_5_ purity of the product reaches 99.05% under the following conditions: precipitation time of 1.5 h, precipitation temperature of 98 °C, initial precipitation pH of 2, ammonium addition coefficient of 2, purification time of 5 min with purification performed twice, purification temperature of 65 °C. In this study, compared with the use of ammonia spirit for vanadium precipitation and ammonium salt vanadium precipitation, the ammonia consumption levels are reduced by 79.80% and 80.00%, and the purity levels are increased by 0.70% and 1.01%, respectively. The compositions of the precipitated (NaV_3_O_8_∙xH_2_O) and purified ((NH_4_)_2_V_6_O_16_·1.5H_2_O) hydrolysis products are characterized via XRD. The TGA results show that NaV_3_O_8_∙xH_2_O contains 1.5 times the amount of crystal water. The FTIR results explain that the two V_3_O_8_^−^ layers are combined end-to-end to form a V_6_O_16_^2−^ layer. The change of the product image indicates that the purification process includes three stages. Firstly, heating and NH_4_^+^ attack expand the V_3_O_8_^−^ layer. NH4^+^ diffuses more easily into the V_3_O_8_^−^ layer. Secondly, NH_4_^+^ destroys the electrostatic interaction between Na^+^ with the V_3_O_8_^−^ layer and replacing Na^+^. Finally, V_3_O_8_^−^ is polymerized into V_6_O_16_^2−^ to keep the crystal structure stable.

## 1. Introduction

Vanadium is a strategic reserve resource [1]. Vanadium pentoxide is one of the most important oxides of vanadium [2], which is a layered amphoteric oxide with good catalytic activity [3]. V_2_O_5_ is widely used in vanadium batteries and electrode materials, nanostructured materials, and optical devices, as well as in metallurgy and the chemical and aerospace industries, owing to its excellent physical and chemical properties [4,5,6,7,8,9]. Due to the widespread application of vanadium pentoxide, its demand and purity requirements are higher. As such, the clean and efficient preparation of high-purity vanadium pentoxide has attracted widespread attention [10].

The vanadium precipitation process is an important step in obtaining vanadium products using vanadium shale extraction technology, which mainly uses the different stability levels of vanadium and impurity ions in the same solution to separate vanadium and impurity ions to obtain high-purity vanadium products [11]. The general process of vanadium precipitation mainly includes hydrolyzed vanadium precipitation, ammonium salt vanadium precipitation, ferric salt vanadium precipitation, and calcium salt vanadium precipitation [12]. Ferric salt and calcium salt vanadium precipitation processes have a small application range and are mainly used to enrich the intermediate products of vanadium [13]. Ammonium salt vanadium precipitation has high efficiency and leads to high product purity [14]. Its ammonium consumption is generally about 8 [12]. However, ammonium salt as a precipitant inevitably produces a large amount of ammonia nitrogen wastewater and ammonia-containing waste gas, which causes a serious threat to the environment [15]. Hydrolyzed vanadium precipitation was widely used in early industrial production. Although the product of the hydrolyzed vanadium precipitation has low purity (around 85.00%), the process results in low levels of environmental pollution and high production efficiency [14]. With the strengthening of environmental protection, the limitations of general vanadium precipitation technology, such as the small application range, low product purity, and environmental problems, seriously restrict the sustainable development of vanadium extraction technology. It is necessary to explore a clean and efficient production technology for V_2_O_5_.

In the process of hydrolyzed vanadium precipitation, the binding capacity of Na^+^ and polyvanadate ions is greater than that of H^+^ [11,14]. Therefore, the vanadate and Na^+^ combine to form sodium polyvanadate and subsequently crystallize. The main reason for the low purity of the product is that a large amount of Na^+^ is precipitated. In the process of ammonium salt vanadium precipitation, the binding capacity of NH_4_^+^ with polyvanadate ion is better than that of Na^+^ [12]. Therefore, ammonium polyvanadate crystals will be precipitated. Due to the different selectivity levels of polyvanadate to cations in the solution, the polyvanadate will precipitate and separate from the most stable structure. Based on this principle, a low-purity sodium polyvanadate precipitate was prepared through the hydrolytic vanadium precipitation process. Due to polyvanadate ion having different selectivity to NH_4_^+^ and Na^+^, the replacement reaction of Na^+^ with NH_4_^+^ may occur during the purification of sodium polyvanadate precipitate with ammonia salt [16]. Then, the high-purity V_2_O_5_ can be prepared by calcining the purified product at a high temperature.

The purpose of this research is to obtain a low-pollution, high-efficiency process by integrating the advantages of multiple vanadium precipitation processes. The combined process can be used to solve the limitations of general vanadium precipitation technologies and environmental problems caused by ammonia nitrogen, and to promote the green development of the vanadium extraction process.

## 2. Experimental

### 2.1. Materials

In this study, all chemical reagents were of analytical grade. All solutions were prepared with deionized water. Vanadium in vanadium-rich liquid is tetravalent. NaClO_3_ as an oxidant oxidizes V(IV) in the vanadium-rich liquid to V(V). Na_2_CO_3_ (Sinopharm Chemical Reagent Co., Ltd, Shanghai, China) was used to adjust the pH of the vanadium-rich solution and ammonium chloride (Sinopharm Chemical Reagent Co., Ltd, China) was used as a detergent. The vanadium-rich liquid in this study was a stripping liquid obtained from vanadium shale (Tongshan, China) through roasting, acid leaching, extraction with D2EHPA (Sinopharm Chemical Reagent Co., Ltd, China), and stripping back extraction with sulfuric acid. Its composition is shown in Table 1. The pH of vanadium-rich liquid was −0.16, as measured using a pH S−3 acidity meter from Shanghai Lida Instrument Factory (Shanghai, China).

### 2.2. Experimental Steps

**Figure 1:**
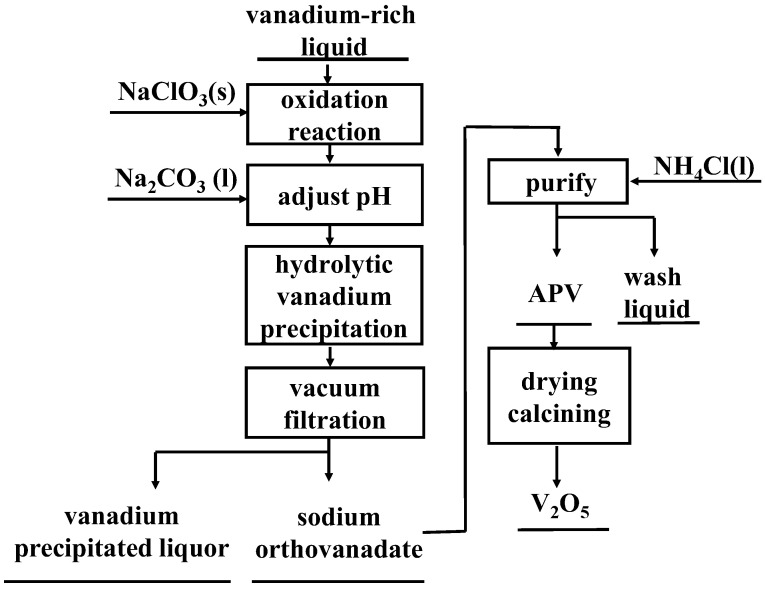
A flow chart of hydrolyzed vanadium precipitation–purification process with ammonium salt.

The process of hydrolytic vanadium precipitation–purification with ammonium salt is shown in Figure 1.

#### 2.2.1. Hydrolyzed Vanadium Precipitation 

The vanadium precipitation efficiency was determined by studying different influencing factors. The experiment was performed as follows. Firstly, NaClO_3_ was added into 30 mL vanadium-rich solution and heated in a water bath to 60 °C for oxidation until the V^4+^ fully oxidized to V^5+^. Secondly, the pH of vanadium-rich liquid after oxidation was adjusted using sodium carbonate until it was suitable for vanadium precipitation, then heating and stirring were performed for a period. Finally, the vanadium precipitation and vanadium precipitation liquor were obtained by solid–liquid separation.

The precipitation efficiency (η) of vanadium was calculated using the following Equation (1):

(1) $$\eta \;=\;1\;-\;\frac{C_{b}V_{b}}{C_{a}V_{a}}\;\times \;100\%$$

where C_a_, C_b_ refer to the vanadium concentrations of vanadium-rich liquid and residual liquid (g/L), respectively; V_a_, V_b_ refer to the volumes of vanadium-rich liquid and residual liquid (L), respectively.

#### 2.2.2. Ammonium Salt Purification 

High-purity vanadium pentoxide was obtained by adjusting the influence of the type and amount of detergent, purification time, purification temperature, and other factors on the purification of the hydrolyzed vanadium product. The specific steps of the purification process were as follows. The ammonium chloride solution with a concentration of 20 g/L was mixed with the hydrolyzed vanadium precipitation product and fully stirred at 450 r/min. The purification process was performed with different ammonium addition coefficients (K), purification frequencies, purification temperatures, and purification times. The purification product was obtained via vacuum filtration after the reaction finished. The product of V_2_O_5_ was calcimined using the purified product at 520 °C for 30 min.

The ammonium addition coefficient K was defined as the ratio of the amount of ammonium ions to the amount of V_2_O_5_ substance in the vanadium-rich liquid. The calculation formula for K was the same as Equation (2):

(2) $$K\;=\;\frac{{n(\mathrm{NH}}_{4}^{+})}{{n(V}_{2}O_{5})}$$

## 3. Characterizations and Analysis

In the study, the vanadium concentration in the vanadium precipitation liquor was measured via ferrous ammonium sulfate titration. The phase composition of the vanadium product was analyzed using an X-ray diffractometer (XRD, D/MAX-RB, Rigaku, Tokyo, Japan) equipped with Cu Kα radiation (λ = 0.15406 nm, 40 KV and 40 KA). Changes in chemical bonds during the reaction were recorded through the Fourier transform infrared (FTIR) spectra (Thermo Fisher Scientific Co., Waltham, MA, USA). The purity of V_2_O_5_ in the product was measured according to YB/T 5328-2009. The chemical element composition in the vanadium pentoxide product was determined by X-ray fluorescence spectrometer (XRF, Netherland PANalytical B.V., AXIOS, Almelo, The Netherlands). The content of crystal water in the product was conducted via thermogravimetric analysis (TG), using an STA449C integrated thermal analyzer from Germany Netzsch (Netzsch, Selb, Germany).

## 4. Results and Discussion

### 4.1. Effects of Hydrolyzed Vanadium Precipitation Conditions on Precipitation Efficiency

**Figure 2:**
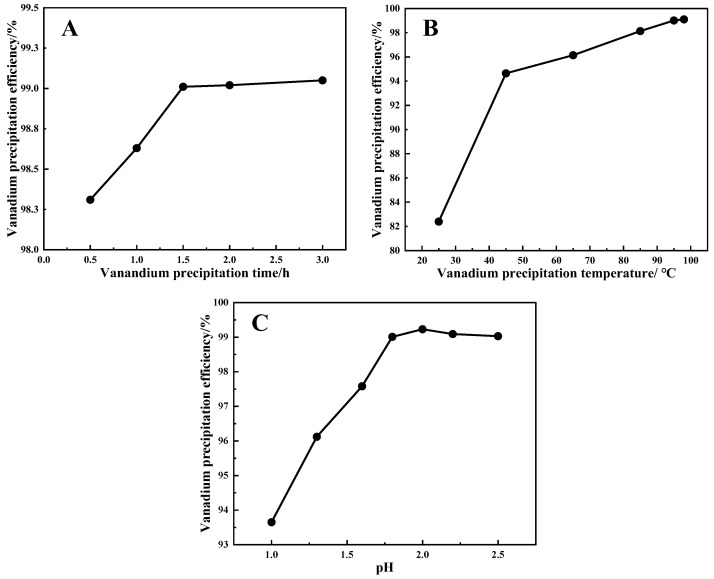
(**A**) Effect of time on the vanadium precipitation efficiency (operation conditions: initial pH of 1.8, temperature of 95 °C). (**B**) Effect of temperature on the vanadium precipitation efficiency (operation conditions: initial pH of 1.8, time of 1.5 h). (**C**) Effect of the initial pH on vanadium precipitation efficiency (operation conditions: time of 1.5 h, temperature of 98 °C).

Figure 2A shows that the vanadium precipitation efficiency can be increased by extending the reaction time, although it does not increase significantly after 1.5 h. This phenomenon indicates that the precipitation reaction reaches equilibrium after 1.5 h. Considering the energy consumption and production efficiency, the optimal time was determined to be 1.5 h in the following experiment.

Figure 2B demonstrates that the temperature significantly influenced the vanadium precipitation efficiency. The hydrolysis of vanadium is difficult at low temperature. The higher the temperature, the more favorable the conditions for vanadium precipitation [17,18]. Due to temperature limitations under normal pressure, 98 °C was selected as the best temperature.

Figure 2C shows that the vanadium precipitation efficiency shows an upward trend due to the dissolution of sodium polyvanadate being reduced [14], and it shows a downward trend owing to the reaction of hydrolyzed vanadium precipitation being weakened [17]. The vanadium precipitation efficiency reaches the maximum value of 99.23% at the initial pH of 2. Therefore, the optimal initial pH for hydrolyzed vanadium precipitation is 2.

The main parameters of the vanadium hydrolysis precipitation process were studied to ensure that as many vanadium ions entered the hydrolysis vanadium precipitation solid product as possible, which is beneficial to the subsequent purification process to prepare more vanadium pentoxide products. Figure 2 reveals that the vanadium precipitation efficiency can reach 99.23% under the following conditions: time of 1.5 h, temperature of 98 °C, initial pH of 2.

### 4.2. Ammonium Salt Purification

#### 4.2.1. Effect of Detergent on Ammonium Salt Purification

**Figure 3:**
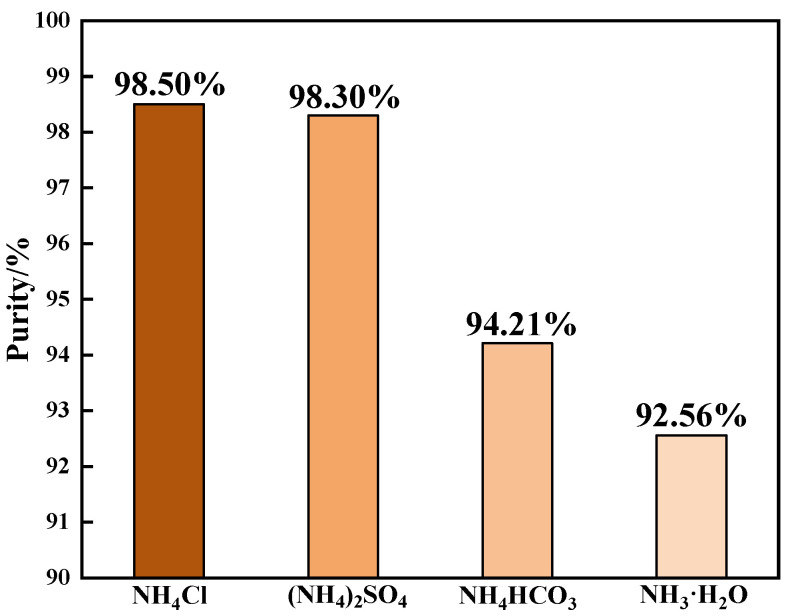
Effects of different detergents on ammonium salt purification (operation conditions: the precipitation temperature was 98 °C, the precipitation time was 1.5 h, the initial pH was 2, the ammonium addition coefficient was 2, the purification temperature was 25 °C with purification performed twice, and the purification time was 5 min).

In this study, the main purpose of choosing ammonium salt as the detergent was to replace Na^+^ with NH_4_^+^ to improve the purity of the product. The general ammonium salts mainly include NH_4_Cl, (NH_4_)_2_SO_4_, NH_4_HCO_3_, NH_3_·H_2_O, and (NH_4_)_2_CO_3_. The production costs are high due to (NH_4_)_2_CO_3_ being expensive. In addition, NH_3_·H_2_O is volatile and inconvenient to store. NH_3_·H_2_O and (NH_4_)_2_CO_3_ are not suitable for actual industrial production. To find the best detergent, the effect of common ammonium salt as detergent on the purification process was studied. The experimental results are shown in the Figure 3.

The tests of the different ammonium salts as detergents were carried out under the following conditions: the temperature was 98 °C, the time was 1.5 h, the initial pH was 2, the ammonium addition coefficient was 2, the purification temperature was 25 °C with purification performed twice, and the purification time was 5 min.

It can be seen from Figure 3 that when NH_4_Cl, (NH_4_)_2_SO_4_, NH_4_HCO_3_, and NH_3_·H_2_O were used as detergents, the V_2_O_5_ purity levels of the purified products were 98.50%, 98.30%, 94.21%, and 92.56%, respectively. The low purity of the products when NH_4_HCO_3_ and NH_3_·H_2_O were used as detergents was due to the alkaline solutions of NH_4_HCO_3_ and NH_3_·H_2_O, as the hydrolysis precipitation vanadium product (sodium polyvanadate) will dissolve into alkaline aqueous solution during the purification process. Cl^−^ can promote the formation of sodium vanadate precipitation, and SO_4_^2−^ will inhibit the precipitation [10]. Therefore, compared with using ammonium sulfate as a detergent, the purity of the vanadium pentoxide product will be higher when ammonium chloride is used as a detergent for purification. The cost of NH_4_Cl was lower than other ammonium salts. Therefore, NH_4_Cl was the best detergent for the purification process.

#### 4.2.2. Effect of Ammonium Addition Coefficient on Product Purity

**Figure 4:**
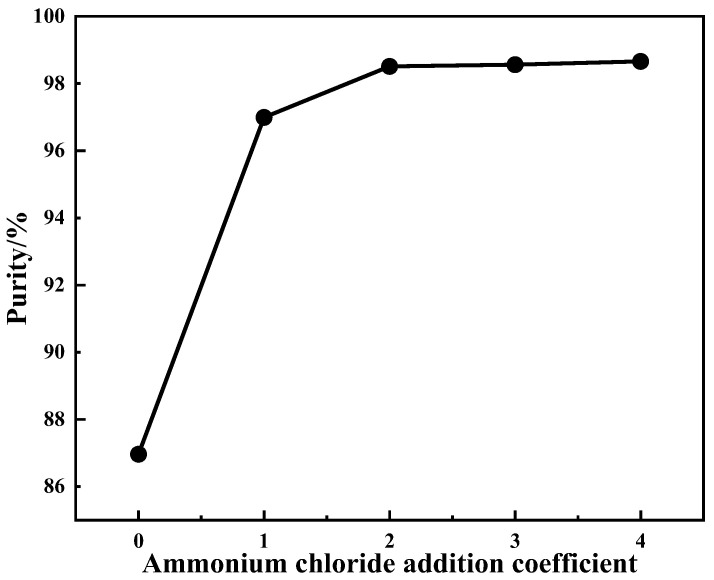
Effect of ammonium addition coefficient on product purity.

The main purpose of the purification process was to reduce ammonium consumption. The influence of different ammonium addition coefficients on the V_2_O_5_ purity of purification products was studied under the following conditions: purification temperature of 25 °C with purification performed twice, purification time of 30 min. The results are shown in Figure 4.

Figure 4 indicates that the purity of the purified product increased with the increase in ammonium addition coefficient. Compared with the hydrolyzed vanadium precipitation product, its purity showed a significant improvement through the ammonium chloride purification. When the ammonium addition coefficient was greater than 2, the purity of the purified product showed no obvious improvement. The product purity was 98.51% at the ammonium addition coefficient of 2. To reduce the ammonium consumption as much as possible while increasing the purity of the purified product, the best ammonium addition coefficient was 2.

#### 4.2.3. Effect of Purification Time on Product Purity

**Figure 5:**
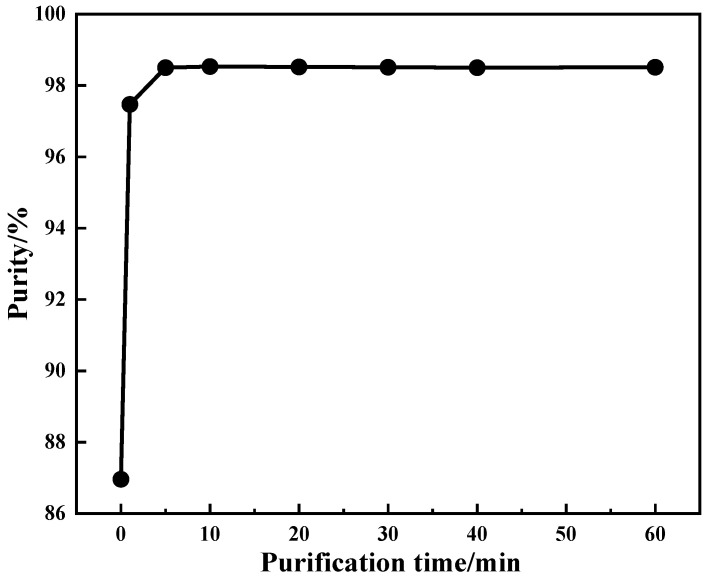
Effect of purification time on product purity.

To investigate the efficiency of the production process, the effect of purification time on the purity of purified products was assessed under the following conditions: purification temperature of 25 °C with purification performed twice, ammonium addition coefficient of 2. The experimental results are shown in Figure 5.

The improvement in product purity was related to the substitution of ammonium for sodium. It can be seen in Figure 5 that the purity of the purified product increases sharply within 0–5 min, which indicates that the purification reaction of ammonium-substituted sodium ions mainly occurs in the first 5 min. After 5 min of purification, the purity of the purified product tends to be constant with the increase in time, which shows that the substitution reaction between ammonium and sodium ions tends to be balanced. Considering the production efficiency and energy consumption, the optimum purification time was 5 min.

#### 4.2.4. Effect of Purification Frequency on Product Purity

**Figure 6:**
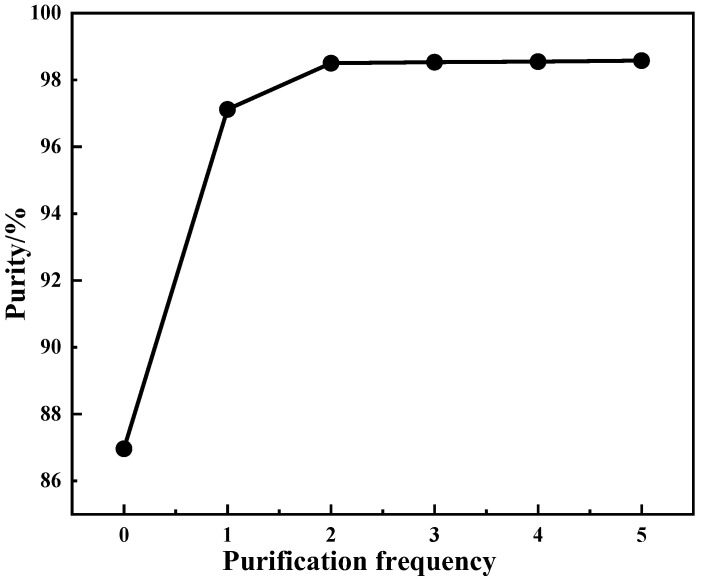
Effect of purification frequency on product purity.

To find the best process conditions, the purity of the purified product should be at least 99%. The purification process was carried out from zero to five times without changing the total ammonium consumption under the following conditions: purification temperature of 25 °C, purification time of 5 min, ammonium addition coefficient of 2. The results are shown in Figure 6.

It can be seen from Figure 6 that the purity of the purified product shows an upward trend with increasing purification frequency increasing. The purity of the purified product increases slowly when the purification frequency exceeds two cycles. This phenomenon indicates that the reaction gradually tends to balance after purification is performed twice. Therefore, to reduce the process flow, improve production efficiency, and reduce energy consumption while ensuring the purity of the purified product, the optimal purification frequency is two cycles.

#### 4.2.5. Effect of Purification Temperature on Product Purity

**Figure 7:**
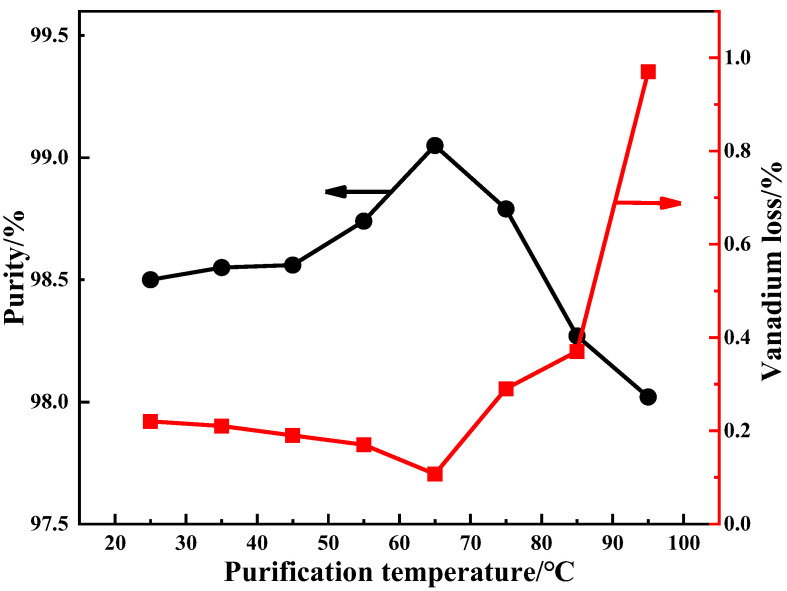
Effect of purification temperature on product purity.

A series of purification tests of the influence of purification temperature on the purification effect were carried out from 25 °C to 95 °C under the following conditions: the purification was performed twice, with a purification time of 5 min and ammonium addition coefficient of 2. The results are shown in Figure 7.

It can be seen in Figure 7 that the purification temperature has a significant effect on the purity of the purified product. As the purification temperature gradually increases from 25 °C to 95 °C, vanadium loss first decreases, then increases. The purity of the purified product shows a trend of rising first and then falling. At the purification temperature of 65 °C, the product reaches a maximum of 99.05%. The replacement of Na^+^ with NH_4_^+^ is easy due to the temperature rises [19]. The binding capacity of NH_4_^+^ with polyvanadate is greater than Na^+^ [12]. Therefore, the V_2_O_5_ purity of purified product will be increased and the vanadium loss will be reduced when the temperature is increased from 25 °C to 65 °C. After the temperature exceeds 65 °C, vanadium loss increases sharply. The dissolution of ammonium polyvanadate will affect the V_2_O_5_ purity of the product [20]. For this reason, 65 °C is the best purification temperature.

### 4.3. Characterization of V_2_O_5_ Product

**Figure 8:**
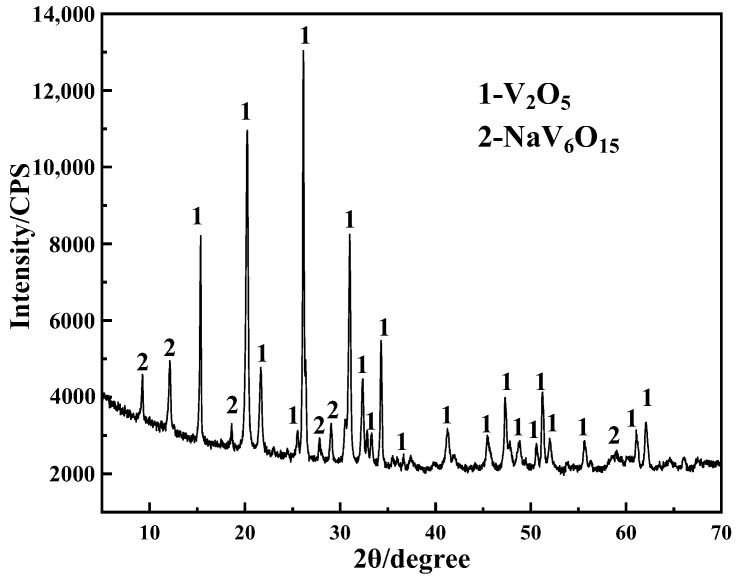
XRD spectra of V_2_O_5_ samples.

Based on the experiments, the vanadium precipitation efficiency can reach 99.23% and the purity of purified product can achieve 99.05% of the value for hydrolytic vanadium precipitation–purification with ammonium salt under the following conditions: time of 1.5 h, temperature of 98 °C, initial pH of 2, ammonium addition coefficient of 2, purification time of 5 min with purification performed twice, purification temperature of 65 °C. The purified product was calcined at 520 °C (V_2_O_5_ product), and the composition of the V_2_O_5_ product is shown in Table 2 and Figure 8.

It can be seen from Figure 8 that the main components of the V_2_O_5_ product are V_2_O_5_ and NaV_6_O_15_. Less miscellaneous peaks of V_2_O_5_ indicate high product purity. Table 2 shows that the content of impurity ions in the V_2_O_5_ product meets the requirements for grade 99 vanadium pentoxide outlined in standard YB/T 5304-2017. Therefore, V_2_O_5_ meeting the standard YB/T 5304-2017 can be successfully prepared via hydrolytic vanadium precipitation–purification using the ammonium salt experimental process.

### 4.4. Comparison of Vanadium Preparation Processes

The general ammonium salt vanadium precipitation process and ammonia spirit precipitation vanadium process were used to perform a test with the vanadium-rich solution in this system under the following conditions: initial pH of 2, time of 1.5 h, and temperature of 98 °C. The experimental results are shown in Table 3.

It can be seen from Table 3 that the vanadium precipitation efficiency reached 99.41%, and the product purity was 98.35% at the ammonium coefficient of K = 9.9 during the ammonia spirit precipitation vanadium process. The ammonium consumption of the hydrolytic vanadium precipitation–purification process with ammonium salt was reduced by 79.80%, and the product purity was increased by 0.70% compared with the ammonia spirit precipitation vanadium process. The vanadium precipitation efficiency was 99.12% and the product purity was 98.04% at K = 10 during ammonium chloride vanadium precipitation. The ammonium consumption of the new combined process was reduced by 80.00%, and the product purity was increased by 1.01% compared with the ammonia chloride precipitation vanadium process. The combined process successfully reduced the ammonium consumption, while ensuring the purity of the product and reducing the environmental pollution of ammonia nitrogen.

### 4.5. Composition and Structure of Purified Products

**Figure 9:**
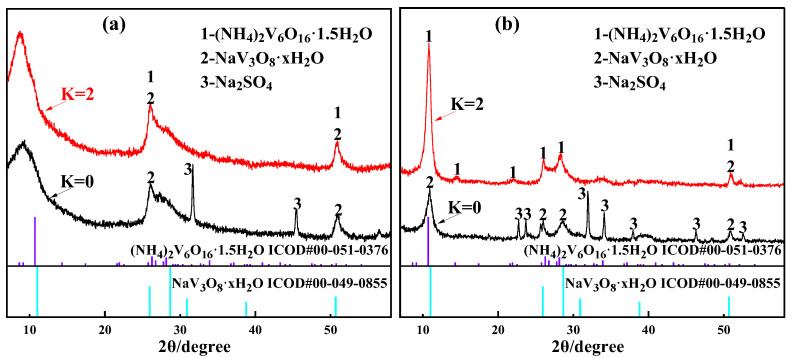
(**a**) XRD spectrum of the product prepared using the vanadium-rich solution containing impurity ions. (**b**) XRD spectrum of the product prepared using the vanadium-rich solution without impurity ions (K = 0—unpurified product; K = 2—purified product).

The transformation of the product composition during the purification process was determined by comparing the phase compositions of the unpurified product (hydrolyzed vanadium precipitation product) and the purified product. Because Al and other impurity ions will affect the crystallization of polyvanadate [14], the compositions of the unpurified product and the purified product were determined using a vanadium-rich solution containing only vanadium (no other impurity ions) to carry out the hydrolytic vanadium precipitation–ammonium salt purification test under the same conditions. The XRD patterns of the unpurified product and purified product obtained under the optimal vanadium precipitation conditions are shown in Figure 9.

A comparison between Figure 9a,b illustrates that amorphous impurity Al ions seriously affect the peak shape of the product. The appearance of the dispersion peak indicates that the crystallinity of vanadium precipitation is not high. Combined with the properties of the vanadium-rich liquid, Al and Na in the precipitation process will reduce the crystallinity of precipitation [12]. Figure 9b proves that the diffraction peaks of the unpurified products (K = 0) correspond to standard peaks of NaV_3_O_8_·xH_2_O (ICOD#00-049-0855). Therefore, the unpurified product (K = 0) comprises NaV_3_O_8_·xH_2_O and Na_2_SO_4_. The excessive sodium content in the crystallized product leads to low purity of the vanadium precipitation product. The diffraction peaks of the purified product (K = 2) correspond to standard peaks of (NH_4_)_2_V_6_O_16_·1.5H_2_O (ICOD#00-051-0376). It can be inferred that the ammonium in the liquid phase enters the purified product. The crystal NaV_3_O_8_·xH_2_O is transformed into (NH_4_)_2_V_6_O_16_·1.5H_2_O crystal during the purification process.

**Figure 10:**
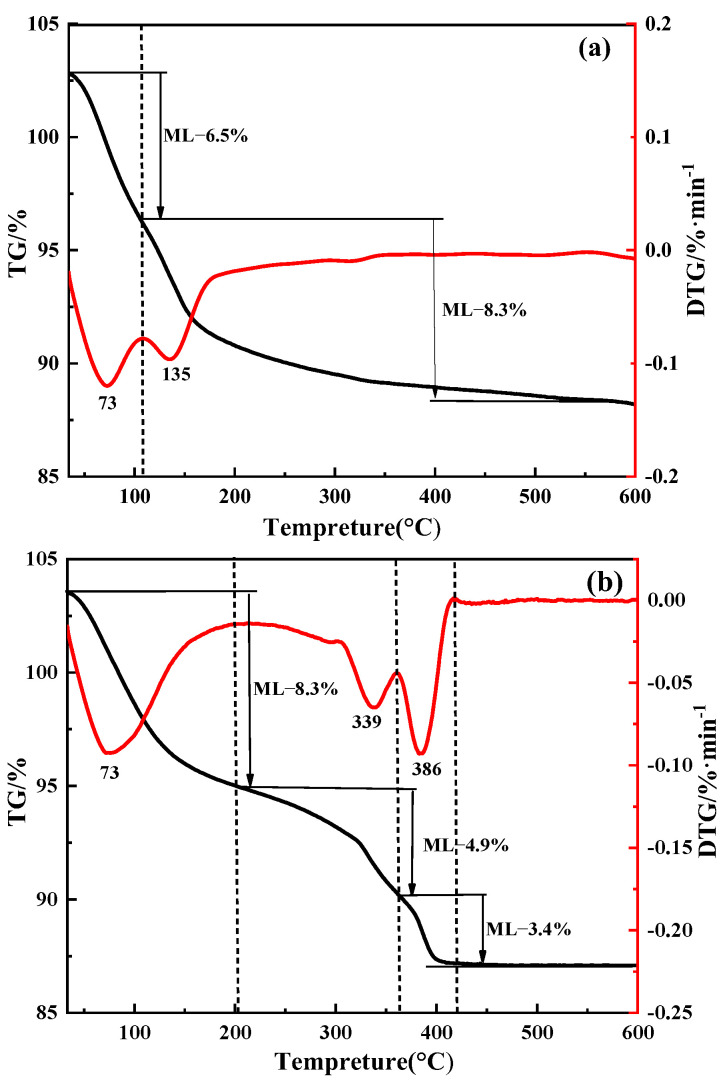
TGA and DTG analyses of purified and unpurified products under nitrogen atmosphere: (**a**) unpurified product; (**b**) purified product.

To further determine the composition of the product, thermogravimetric analysis (TGA) and derivative thermogravimetry (DTG) analyses were performed to further investigate the presence of the product. The results are shown in Figure 10.

Figure 10a shows the two weight loss ranges of unpurified products. The first weight loss range was from 34 to 108 °C, and the second weight loss was from 108 to 600 °C [21]. The thermal decomposition rate reached its maximum at 73 °C and 135 °C. The first weight loss of ≈6.5% for unpurified products corresponded to the evaporation of water. The second weight loss of ≈8.3% occurred due to the departure of structural water, which was intercalated between the vanadate layers. One can calculate that NaV_3_O_8_·xH_2_O crystal contains 1.5 times the amount of crystal water by calculating the weight loss in the second stage. Therefore, the chemical formula for the unwashed products can be determined as NaV_3_O_8_∙1.5H_2_O. Figure 10b indicates the three weight loss ranges of purified product. The thermal decomposition rates reach their maximum values at 73 °C, 339 °C, and 386 °C, respectfully. The first weight loss of ≈8.3% from 34 to 207 °C for purified product corresponded to the evaporation of weakly adsorbed structural water [22]. The second weight loss of ≈4.9% from 207 to 360 °C was attributed to the decomposition of (NH_4_)_2_V_6_O_16_·1.5H_2_O and the third weight loss of ≈3.4% from 360 to 408 °C corresponded to the deintercalation of strongly bound H_2_O via Equation (3) [18,23]. It can be determined that the composition of the unpurified product is NaV_3_O_8_∙1.5H_2_O and the purified product is (NH_4_)_2_V_6_O_16_∙1.5H_2_O. The component of the purified product is (NH_4_)_2_V_6_O_16_ · 1.5H_2_O as indicated by the literature comparison. The results of TG and XRD analyses are consistent:

(NH_4_)_2_V_6_O_16_∙1.5H_2_O → 3V_2_O_5_ + 2NH_3_↑ + 2.5H_2_O (3)

**Figure 11:**
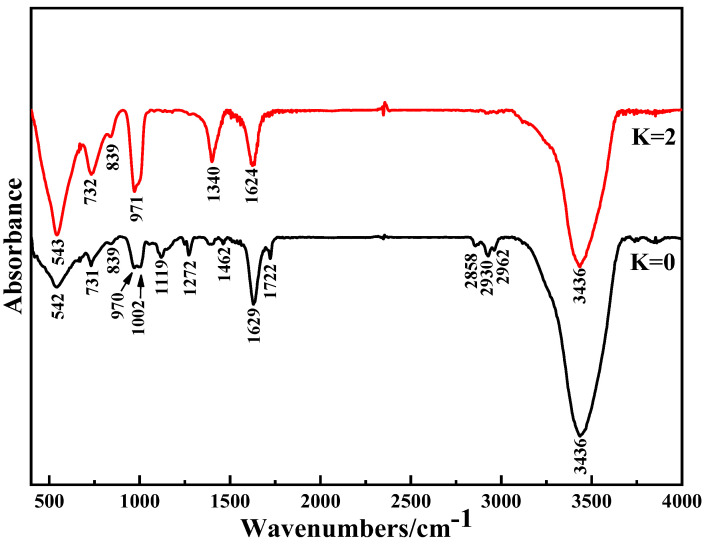
FTIR spectra of unpurified and purified products (K = 0−unpurified product; K = 2−purified product).

Through phase and thermogravimetric analyses, chemical reactions related to NH_4_^+^ during the purification process were identified. NH_4_^+^ in the liquid phase enters the purified product and exists as (NH_4_)_2_V_6_O_16_·1.5H_2_O crystals. To evidence the chemical changes and reveal the mechanism of NH_4_^+^ in the purification process, the products with ammonium addition coefficients of K = 0 (unpurified product) and K = 2 (purified product) were analyzed via FTIR. The analysis results are shown in Figure 11.

It can be seen from Figure 11 that the peaks at 542 cm^−1^ and 543 cm^−1^ are attributed to symmetric stretching vibration of V−O−V [24,25], while the peaks at 731 csm^−1^ and 732 cm^−1^ correspond to the asymmetric V−O−V bending mode [26,27]. The characteristic peaks of the two types of V−O−V are shifted from low to high peaks after the purification process, which means that the intervention of NH_4_^+^ will influence the structure of the NaV_3_O_8_·1.5H_2_O crystal. The V_3_O_8_^−^ layers exist due to the peak at 839 cm^−1^ being assigned to the symmetric stretching mode of the VO_6_ [28]. The double peak of the terminal V=O bond at 970 cm^−1^ and 1002 cm^−1^ disappears [29,30], and turns into a single peak of the V−O−V bond at 971 cm^−1^ [31], which illustrates that the two V_3_O_8_^−^ layers will be combined end-to-end and form a V_6_O_16_^2−^ layer. The peak at 1400 cm^−1^ could be ascribed to NH_4_^+^ bending vibration absorption, which could prove that NH_4_^+^ exists in the purified product as (NH_4_)_2_V_6_O_16_·1.5H_2_O crystals [32].

#### Crystal Purification Mechanism

The phase analysis shows that the NaV_3_O_8_·1.5H_2_O crystal is a layered compound [33]. The V_3_O_8_^-^ layer of NaV_3_O_8_·1.5H_2_O crystal is composed of VO_6_ octahedrons and VO_5_ square pyramids sharing a vertex oxygen atom. Na^+^ is in the interlayer octahedral gap of V_3_O_8_^−^ layers. The layer spacing (d_1_) of the V_3_O_8_^−^ layer is 7.08 Å [34]. The positively charged Na^+^ and the V_3_O_8_^−^ layer are combined by electrostatic force [35]. The structure of (NH_4_)_2_V_6_O_16_·1.5H_2_O crystal is similar to NaV_3_O_8_·1.5H_2_O crystal. The layer spacing (d_2_) of the V_6_O_16_^−^ layer is 8.191 Å [36]. The hydrogen atoms in NH_4_^+^ combine with adjacent oxygen atoms of the V_6_O_16_^−^ layer via the hydrogen bond [37].

**Figure 12:**
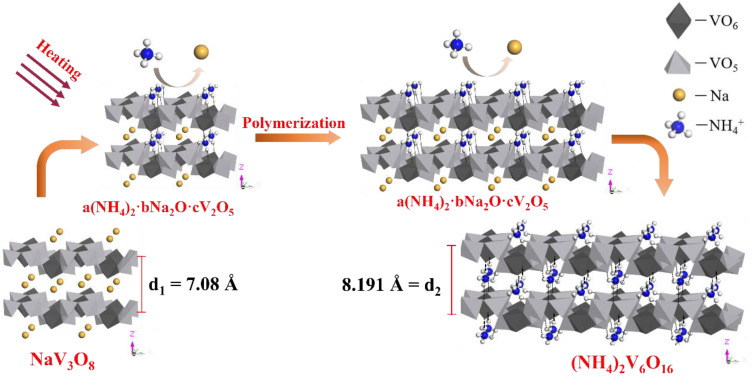
Schematic diagram of the probable mechanism of the purification process.

Combining the experimental phenomena and analyses shows that the purification process probably includes the replacement of Na^+^ with NH_4_^+^ and the polymerization of V_3_O_8_^−^ into V_6_O_16_^2−^. The structures of the NaV_3_O_8_ crystal and (NH_4_)_2_V_6_O_16_ crystals are used to describe the transformation mechanism of the purification process. The probable mechanism of the purification process is shown in Figure 12.

The probable mechanism of the purification process is shown in Figure 12. The entire purification process mainly involves three phases. Firstly, the V_3_O_8_^−^ layer spacing of NaV_3_O_8_·1.5H_2_O crystals will expand via heating [38]. The binding capacity of NH_4_^+^ and polyvanadate is greater than Na^+^ [12], which will make the diffusion and migration of NH_4_^+^ in the V_3_O_8_^-^ layer easier [19]. Secondly, NH_4_^+^ will attack the Na^+^ of the NaV_3_O_8_·1.5H_2_O crystal after the intervention of NH_4_^+^. The electrostatic combination of Na^+^ with the V_3_O_8_^−^ layer will be destroyed. NH_4_^+^ will replace Na^+^ in the octahedral vacancy in the V_3_O_8_^−^ layer [35]. The intermediate product (a(NH_4_)_2_∙bNa_2_O∙cV_2_O_5_, where a, b, and c are related to reaction conditions) is a kind of crystal that includes Na^+^ and NH_4_^+^ [14]. The hydrogen atoms in NH_4_^+^ will combine with adjacent oxygen atoms on the V_3_O_8_^−^ layer through intermolecular hydrogen bonds [39]. Finally, the pH of 20 g/L ammonium chloride solution is 4.23. The aggregated state of vanadium mainly exists in the form of V_6_O_16_^2−^ in the pH range of 2.2–5.0 [40]. The destruction of that layered structure also creates conditions for polymerization. Therefore, the polymerization of V_3_O_8_^−^ to V_6_O_16_^2−^ occurred. The most stable (NH_4_)_2_V_6_O_16_·1.5H_2_O crystal was formed through continuous replacement reaction and polymerization reaction.

## 5. Conclusions

(1) During the hydrolytic vanadium precipitation–purification process with ammonium salt, 99.23% vanadium precipitation efficiency and 99.05% V_2_O_5_ purity can be achieved under the following conditions: time of 1.5 h, temperature of 98 °C, initial pH of 2, ammonium addition coefficient of 2, purification time of 5 min with purification performed twice, purification temperature of 65 °C. The purity of the purified product meets the standard YB/T 5304-2017.

(2) The ammonium consumption during the hydrolytic vanadium precipitation–purification process with ammonium salt was reduced by 79.80% compared with the ammonia spirit vanadium precipitation process and reduced by 80.00% compared with the ammonium chloride vanadium precipitation process. While the combined process reduces ammonium consumption, the purity of vanadium products is improved to above 99.05%. The process reduces the generation of ammonia nitrogen wastewater and makes the preparation of V_2_O_5_ more environmentally friendly.

(3) The mechanism of the ammonium salt purification process was revealed. The purification process can be divided into three reaction stages. First, the V_3_O_8_^−^ layers of sodium vanadate crystal is enlarged via heating and the attack of NH_4_^+^, which make it easier for the NH_4_^+^ to diffuse into the V_3_O_8_^−^ layer. Secondly, NH_4_^+^ destroys the electrostatic bond between Na+ with the V_3_O_8_^−^ layer and replaces the Na^+^ in the octahedral vacancy of the V_3_O_8_^-^ layer. Finally, the polymerization of V_3_O_8_^−^ to V_6_O_16_^2−^ occurs due to the aggregated state of vanadium mainly existing in the form of V_6_O_16_^2−^ in the pH range of 2.2–5.0.

## Figures and Tables

**Table 1:** Chemical composition of the vanadium-rich liquid (g/L).

Element	V	K	Ca	Na	Mg	Al	Si	P	As	Fe
Content	42.27	0.37	0.44	0.42	0.08	8.80	0.26	0.48	0.37	0.12

**Table 2:** Chemical composition of V_2_O_5_ sample (%).

Item	V_2_O_5_	Si	Fe	P	S	As	Na_2_O + K_2_O
V_2_O_5_ sample	99.05	<0.01	0.075	0.026	0.01	<0.01	0.74
Standard sample	≥99.00	≤0.08	≤0.08	≤0.03	≤0.08	≤0.01	≤0.8

**Table 3:** Comparison of different vanadium precipitation processes.

Item	Precipitation by NH_3_·H_2_O	Precipitation by NH_4_Cl	Purification by NH_4_Cl
K	9.9	10	2.0
efficiency	99.41%	99.12%	99.23%
purity	98.35%	98.04%	99.05%

## Data Availability

No new data were created or analyzed in this study. Data sharing is not applicable to this article.
